# Dental Health Utilization in the Republic of Palau: A Survey to Determine Feasibility of an Oral Cancer Screening Program

**DOI:** 10.1200/GO.22.42000

**Published:** 2022-05-05

**Authors:** Katherine Rieth, Timothy Dye, Edolem Ikerdu, Scott McIntosh, Angela Sy

**Affiliations:** ^1^ University of Rochester Medical Center, Rochester, NY; ^2^ Palau Ministry of Health, Koror, Palau; ^3^ University of Rochester, Rochester, NY; ^4^ University of Hawai'i, Honolulu, HI

## Abstract

Oral cancer is the 5th most common cancer in Palau. Betel nut use is a highly prevalent cultural tradition in Palau and also a carcinogen, whose effect may be synergistic with the addition of smokeless tobacco in betel quid in the development of oral cancer. Prognosis can be improved with early detection. The purpose of this study is to assess the feasibility of an oral cancer screening program using existing dental health infrastructure with mixed methods assessment of utilization of dental health resources by adults in Palau.

**METHODS:**

An Internet-based survey was used. Both quantitative and qualitative assessments of dental health care utilization were explored. Quantitative analyses of survey responses were done using descriptive and analytical statistical functions. Open-ended survey questions were coded and analyzed for emergent themes to develop recommendations for the proposed intervention.

**RESULTS:**

The survey was available (Facebook Ads) for 9 days. There were 223 completed surveys, age range was 18-75 (mean = 42.7 years), 177 (80.1%) identified as female, 60.4% reside on Koror, and 60.4% have some university/college level education. Dental care was seen to be important (mean score 82.27). Most felt there were no major barriers to seeing a dentist (n = 164, 72.5%). Themes pertaining to barriers to access focused on lack of availability of appointments and cost. Themes pertaining to facilitators included multilevel resources and services provided by dental clinics.

**CONCLUSION:**

Dental care is important to Palauans, and most would prefer to have this occur in a clinic setting. The themes generated suggest that while there are important facilitators that are in place to promote seeking and obtaining dental care, the existing infrastructure and access to dental care currently do not support an oral cancer screening program. However, these data do provide important areas to address the delays associated with dental care that can improve access and support the implementation of oral cancer screening in the future.

